# Zika virus: Epidemiological surveillance of the Mexican Institute of Social Security

**DOI:** 10.1371/journal.pone.0212114

**Published:** 2019-02-11

**Authors:** Concepción Grajales-Muñiz, Víctor Hugo Borja-Aburto, David Alejandro Cabrera-Gaytán, Teresita Rojas-Mendoza, Lumumba Arriaga-Nieto, Alfonso Vallejos-Parás

**Affiliations:** 1 Division of Epidemiological Surveillance of Communicable Diseases, Instituto Mexicano del Seguro Social (IMSS), Ciudad de México, México; 2 Unit of Primary Health Care, IMSS, Ciudad de México, México; Faculty of Science, Ain Shams University (ASU), EGYPT

## Abstract

**Introduction:**

At the end of 2015, the first cases of Zika were identified in southern Mexico. During 2016, Zika spread as an outbreak to a large part of the country's coastal zones.

**Methodology:**

The Zika epidemiological surveillance system records cases with clinical symptoms of Zika virus disease (ZVD) and those confirmed by means of a reverse polymerase chain reaction (RT-PCR) assay. This report includes the suspected and confirmed cases from 2016. Incidence rates were estimated by region and in pregnant women based on the proportion of confirmed cases.

**Results:**

In total, 43,725 suspected cases of ZVD were reported. The overall incidence of suspected cases of ZVD was 82.0 per 100,000 individuals and 25.3 per 100,000 Zika cases. There were 4,168 pregnant women with suspected symptoms of ZVD, of which infection was confirmed in 1,082 (26%). The estimated incidence rate of ZVD for pregnant women nationwide was 186.1 positive Zika cases per 100,000 pregnant women.

**Conclusions:**

The incidence of Zika in Mexico is higher than that reported previously in the National System of Epidemiological Surveillance. Positive cases of Zika must be estimated and reported.

## Introduction

Zika virus (ZIKV) was originally identified in a sentinel rhesus monkey in the Zika Forest of Uganda in 1947. The virus is a member of the family Flaviviridae, genus *Flavivirus*, and is mainly transmitted to humans by *Aedes* genus of mosquitoes [1]. The first recorded outbreak of Zika virus disease (ZVD) was reported on the Island of Yap (Federated States of Micronesia) in 2007 [2], where approximately three quarters of Yap residents were infected with Zika virus [3]. This was followed by a large outbreak of ZIKV infection in French Polynesia between October 2013 and April 2014; during the same period, an increase in Guillain-Barré syndrome was reported, suggesting a possible association between ZIKV and Guillain-Barré [4].

The outbreak of ZVD started in Brazil in 2015 [5], and an increased number of reported cases of microcephaly was also reported [6]. Subsequently, ZIKV was found in fetal brain tissue [7]. Currently, a congenital Zika syndrome is recognized that involves a spectrum of changes, including other manifestations of neurological and fetal development [8].

As of August 25, 2017, 48 countries and territories of the Americas have reported more than 554,479 suspected cases of ZVD, including 207,557 confirmed cases of autochthonous transmission [9].

In Mexico, the presence of the virus has been documented since January 2015 [10], and the first laboratory-confirmed autochthonous cases of ZVD in humans were identified in October 2015 [11]. Although many cases were estimated for the region at the beginning of the epidemic, the magnitude of the epidemic has not been precisely determined, since only confirmed cases of ZIKV are published, and not all suspected cases are confirmed in the laboratory [12]. A recent study in Mexico estimated that the number of symptomatic cases of Zika infection in the general population was 7.3 times greater than the corresponding number of reported cases [13].

In Mexico, 46 cases of congenital syndrome associated with Zika have been confirmed between 2016 to 2018, and 19 confirmed cases of Guillain-Barré syndrome were associated with Zika [14–15].

Epidemiological surveillance was performed through the National Epidemiological Surveillance System (SINAVE) [16]. The Mexican Ministry of Health maintains a national public health surveillance system for notifiable conditions, including surveillance of vector-borne diseases (VBDs), such as dengue, chikungunya and ZVD. The epidemiological surveillance is based on operational definitions of the conditions that are applied by all institutions of the National Health System (including both social security institutions and others) [17]. Information that was collected by health care centers was compiled and transmitted to the National Public Health surveillance system, which publishes the confirmed cases of ZVD weekly.

The Mexican Social Security Institute (IMSS) is the largest institution of social security in the country that provides medical services. As part of the National Mexican Health System, the IMSS is actively involved in the epidemiological surveillance systems and covered 53,300,386 insured individuals in 2016. The aim of this study was to characterize the epidemiology of ZVD in Mexico. We describe the incidence of ZVD according to age, geographic distribution, and pregnancy after the first year of the outbreak in Mexico.

In this study, we also describe the epidemiological surveillance results, including all suspected and confirmed symptomatic cases reported in the IMSS, to estimate the cumulative incidence rates of ZVD.

## Methods

This descriptive analysis is part of a research project that was approved by the Institutional Review Board of the IMSS (approval number R-2016-785-076).

No consent was needed because all data were fully anonymized before we had access to them. This report includes all reported cases of ZVD in the IMSS surveillance system from January 3 to December 31, 2016.

The epidemiological surveillance system uses the following definitions for ZVD.

A suspected ZVD case is defined as a patient with cutaneous exanthema with two or more of the following signs or symptoms: fever, headache, conjunctivitis (not purulent/hyperemic), arthralgia, pruritus or retroocular pain and any epidemiological association.

Epidemiological association was defined as the presence of the *Aedes aegypti* or *Aedes albopictus* vector and previous visit or residence in areas of transmission in the two weeks prior to the appearance of clinical signs or the existence of confirmed cases in the locality. In addition, an individual with a history of unprotected sexual contact within two weeks prior to the onset of symptoms with a person who has a history of residency or travel to an area with local transmission of ZIKV or with presence of vectors in the 8 weeks prior to sexual contact was also defined as a suspected case.

A confirmed case of ZVD was defined as a suspected case of ZVD who was positive for ZIKV by detecting viral RNA via real-time RT-PCR in blood serum samples taken within the first five days of clinical onset (fever and/or other symptoms).

Molecular detection of ZIKV was performed by real-time RT-PCR uniplex. We amplified the 77-bp fragment with the following primers for characterization of the coding region of the viral E protein gene: CCGCTGCCCAACACAAG; FAM–AGCCTACCTTGACAAGCAGTCAGACACTCAA–BHQ-1; CCACTAACGTTCTTTTGCAGACAT [18] according to the Lanciotti protocol. The sensitivity for the test is 25 genomic copies per reaction. The thermal profile consisted of a reverse transcription step at 50°C for 30 minutes, activation of the enzyme at 95°C for 15 minutes, followed by 45 cycles of 95°C for 15 seconds and 60°C for 1 minute for hybridization and extension using a 7500 Fast Real-Time PCR system from Applied Biosystems. (Foster City, California, USA).

National standards for laboratory epidemiological surveillance do not include serological tests, such as for IgM or IgG.

Laboratory sampling was performed using the following criteria: in localities where the circulation of ZIKV had not been identified, blood serum samples were taken to detect viral RNA by real-time RT-PCR from 100% of cases that met the definition of a suspected ZVD case, regardless of the condition of the patient. Once viral circulation was identified by a positive real-time RT-PCR result in the locality, a blood serum sample was randomly obtained from 5% of cases that met the operative definition of a suspected case of ZVD (S1 Fig).

In the National Epidemiological Surveillance System, there are local legal committees where all health institutions meet to assess the monthly viral circulation of Zika.

For pregnant women who met the operative definition of a suspected ZVD case, samples were obtained from 100% of cases.

All patient samples were processed by laboratories authorized by the Institute of Epidemiological Diagnosis and Reference (InDRE), which is the National Reference Laboratory of the country [19].

The incidence rates were estimated using the suspected cases of ZVD (including pregnant women) as the numerator and the population insured by IMSS as the denominator (as of June 2016, i.e., the population insured in the middle of the period). To determine the incidence rate in pregnant women, notified symptomatic pregnant women were used as the numerator and the number of total pregnant women in the IMSS as the denominator (based on the annual census of pregnant women from January 3 to December 31, 2016).

Given that laboratory samples were taken from 5% of suspected cases once virus circulation was determined by epidemiological surveillance, it was necessary to estimate the positive cases based on the sampling of 5% of the cases.

To estimate positive cases of ZVD, the suspected ZVD cases without a laboratory sample were multiplied by the positive plus the confirmed cases.

To calculate the proportion of positive ZVD cases, we divided the number of PCR-positive ZVD cases by the suspected cases of ZVD.

Positivity is the probability of suffering ZVD among people suspected of the disease who did not have a laboratory sample. Positivity was obtained by dividing the positive cases by the sum of positive and negative cases (the total number of cases with a laboratory sample). The positivity was obtained per epidemiological week and state (region).

The data were stratified by regions of the country: 1) center, 2) northeast and 3) southeast, according to the National Health and Nutrition Survey of 2012 [20]. The regions were formed by a group of states as follows: 1) Center: Aguascalientes, Colima, Guanajuato, Jalisco, México, Michoacán, Morelos, Nayarit, Querétaro, San Luis Potosí, Zacatecas and Mexico City; 2) Northern: Baja California, Baja California Sur, Coahuila, Chihuahua, Durango, Nuevo Leon, Sinaloa, Sonora and Tamaulipas; 3) Southern: Campeche, Chiapas, Guerrero, Hidalgo, Oaxaca, Puebla, Quintana Roo, Tabasco, Tlaxcala, Veracruz and Yucatán.

Pregnant women were classified according to trimester. The first trimester was defined as 1 to 12 weeks of gestation, the second as 13 to 28 weeks of gestation and the last as 29 to 42 weeks of gestation, according to the Office on Women's Health at the US Department of Health and Human Services [21].

A chi-squared hypothesis test was used to determine the statistical association in suspected cases of ZVD between hospitalization and comorbidities and pregnancy.

## Results

### Notified cases of ZVD

**Fig 1:**
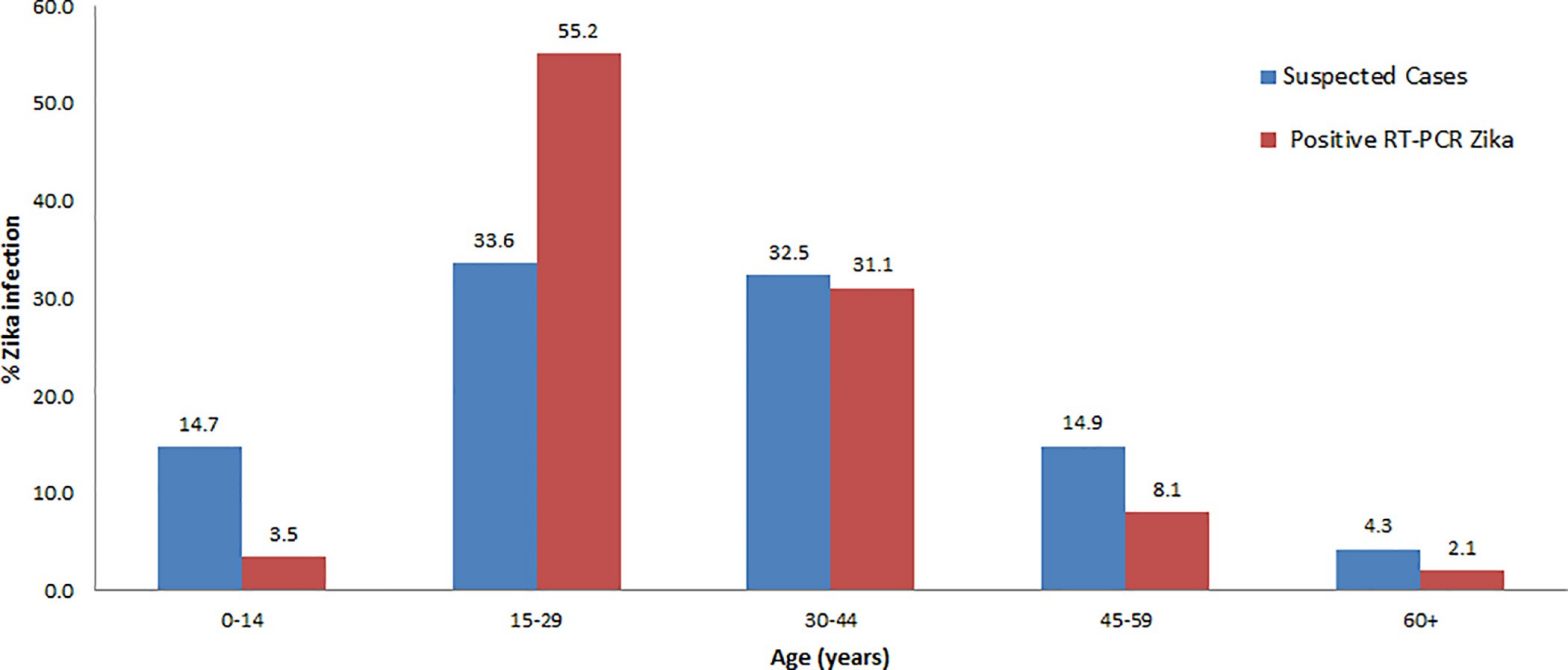
Age distribution of Zika infection in Mexico, 2016. Suspected cases in total 43,725. Positive cases by RT-PCR in total 1,700.

From January 3 to December 31, 2016, 43,725 suspected cases of ZVD were reported in the IMSS. Of these cases, 5,676 (13%) had a suitable sample for RT-PCR for ZIKV identification. Of these cases, 1,700 were positive by RT-PCR for ZIKV, with a mean period of positivity of 0.32. We estimated a total of 13,487 positive cases, which is 30.8% of the suspected cases. The median age of the suspected ZVD cases was 30 years and was 28 years for cases positive by RT-PCR. The interquartile range was 19 years for suspected cases and 11 years for positive cases. In total, 63.7% of the suspected cases were women, and 88.2% of the positive cases emphasized monitoring pregnant women for associations of defects in babies. Fig 1 shows the data by age group, where there is an elevation in the age group 15–44. The confirmed Zika cases were more elevated in the 15–29 age group.

The overall incidence of suspected ZVD cases was 82.0 per 100,000. This value was 99.4 in women (including pregnant women), 86.3 per 100,000 in nonpregnant women, and 62.8 per 100,000 in men.

Based on age group, the highest incidence was recorded in individuals who were 20 to 24 years of age, in both sexes, with an incidence rate of 169.8 per 100,000. This value was 219.6 in women and 119.7 in men. (Table A in S1 File)

The main clinical symptoms presented by the suspected cases of ZVD included rash in 41,808 (95.6%) and pruritus in 36,788 (84.1%) cases, findings that are also reflected in the cases confirmed by PCR (Table B in S1 File).

Statistical associations were found between hospitalization for suspected ZVD cases and diabetes (odds ratio (OR) 3.1, confidence interval (CI) 2.2–4.3), hypertension (OR 2.3, CI 1.7–3.1) and pregnancy (OR 4.7, CI 4.0–5.6), all with a p-value = 0.001.

Two patients with a negative result for ZIKV by RT-PCR died; the two cases were hospitalized.

The first case was a female infant with a severe acute condition, and the date of sampling was performed four days after the onset of clinical symptoms.

The second patient was a male with HIV infection; he died of a pneumonic episode 18 days after the onset of ZVD clinical symptoms.

**Fig 2:**
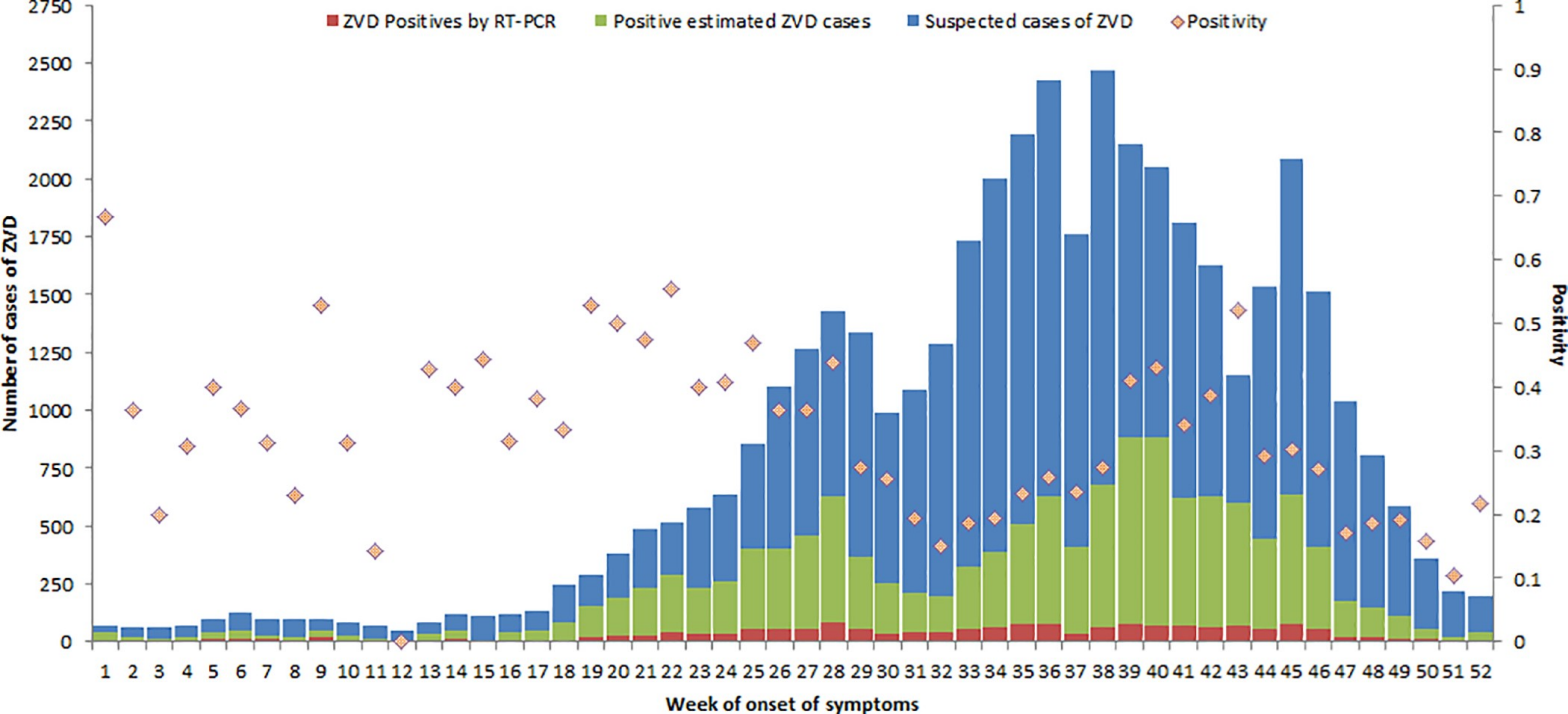
Epidemic curve of suspected cases of ZVD, positive cases determined by RT-PCR and positive estimates cases at IMSS, 2016. ZVD = Zika Virus Disease. Positivity is the probability of suffering ZVD among people suspected of the disease who did not have a laboratory sample.

The weekly variation (2016) in the numbers of suspected, confirmed and estimated Zika cases is presented in Fig 2, which includes all reported cases (including pregnant women) by epidemiological week at the national level of the IMSS.

The number of reported cases of ZVD increased from May to October. The highest number of cases was reported during week 38 (from September 18 to 24). After that week, the number of cases decreased overall. Regarding the positive estimated cases, the epidemiological curve revealed a climax at the end of September and the beginning of October, with a maximum record at week 39 of 883 cases (Table C in S1 File).

All states of the country reported suspected cases of ZVD to the IMSS. In total, 78.9% of suspected ZVD cases and 76.2% of confirmed cases were reported in the southeastern region of the territory. The total rate of positive estimated cases was 25.3 per 100,000 among the estimated positive cases Table 1.

**Table 1:** Estimated positive cases and estimated positive rate by state. IMSS, 2016.

State	IMSS insured population in the middle of the period (June 2016)	Estimated positive cases of ZVD	Incidence rate of estimated positive cases of ZVD per 100,000
Aguascalientes	850,789	1	0.1
Baja California	2,109,338	0	0.0
Baja California Sur	440,768	8	1.7
Campeche	324,936	484	149.0
Coahuila	2,438,396	75	3.1
Colima	411,056	925	225.0
Chiapas	655,081	2,344	357.8
Chihuahua	2,426,800	0	0.0
Durango	854,451	0	0.0
Guanajuato	2,558,267	0	0.0
Guerrero	630,886	2,245	355.8
Hidalgo	755,240	85	11.2
Jalisco	4,617,581	90	2.0
México	6,395,052	24	0.4
Michoacán	1,359,919	11	0.8
Morelos	763,285	69	9.0
Nayarit	497,291	27	5.5
Nuevo León	3,925,994	1,020	26.0
Oaxaca	611,255	884	144.6
Puebla	1,549,815	13	0.8
Querétaro	1,232,440	3	0.2
Quintana Roo	839,337	344	41.0
San Luis Potosí	1,181,468	27	2.2
Sinaloa	1,662,910	68	4.1
Sonora	1,687,518	19	1.1
Tabasco	565,761	650	114.9
Tamaulipas	1,984,239	235	11.8
Tlaxcala	367,293	2	0.5
Veracruz	2,567,287	1,183	46.1
Yucatán	1,021,733	3,200	313.2
Zacatecas	558,023	0	0.0
Mexico City	5,456,180	49	0.9
**Total IMSS**	**53,300,386**	**13,487**	**25.3**

### Pregnant women ZVD cases

From January 3 to December 31, 2016, the IMSS registered 4,168 suspected cases of ZVD in pregnant women (9.5% of total cases). Positive results by PCR were noted in 1,082 cases (26.0%). The median age of women with ZVD was 27 years, with an interquartile range (IQR) of 8 years Table 2.

**Table 2:** Characteristics of pregnant women Zika cases in the Mexican Institute of Social Security, 2016.

Characteristic	Suspected cases		Positive cases by RT-PCR	
	N = 4,168		N = 1,082	
	Number	(%)	Number	(%)
**Age group**				
0–14	8	0.2	2	0.2
15–29	2844	68.2	754	69.7
30–44	1310	31.4	326	30.1
45–59	6	0.1	0	0
**Trimester of symptom onset**				
First trimester	970	23.3	219	20.2
Second trimester	1962	47.1	628	58.0
Third trimester	1236	29.7	235	21.7
**Region**				
Northern region	383	9.2	128	11.8
Central region	311	7.5	69	6.4
Southern región	3474	83.3	885	81.8
**Clinical signs**				
Exanthema	3937	94.5	1044	96.5
Pruritus	3411	81.8	889	82.2
Headache	3045	73.1	776	71.7
Myalgia	2903	69.6	729	67.4
Arthralgias	2826	67.8	701	64.8
Conjunctivitis	2571	61.7	655	60.5
Fever	2059	49.4	509	47.0
Retroocular pain	1600	38.4	406	37.5
Diarrhea	436	10.5	109	9.5
**Other features**				
Hospitalizade	237	5.7	53	4.9
Diabetes Comorbidity	20	0.5	3	0.3
Hypertension Comorbidity	31	0.7	7	0.6
Indigenous ethnicity	26	0.6	11	0.2

Laboratory samples were taken from 3,390 pregnant women (81.3% of all pregnant women suspected cases). From these samples, 1,082 pregnant women were found to be positive for ZVD by RT-PCR (31.9%); 1,522 samples were negative for ZVD by RT-PCR (44.9%); and 786 (23.1%) samples could not be processed due to laboratory rejection criteria for biological samples, including lipemic, contaminated or hemolysed samples; samples with insufficient volume to perform the RT-PCR; samples with incomplete information, incorrect data, illegibility, or insufficient quantity; or samples that were not kept consistently cold prior to analysis. Samples that were not processed by RT-PCR were considered suspected cases. Of the total laboratory samples with a result (2,604), 41.5% of pregnant women were positive for ZVD by PCR.

Of the PCR-positive pregnant women, 58.0% were infected by Zika in the second trimester of pregnancy.

The incidence of suspected ZVD in pregnant women nationwide was 717 per 100,000 pregnant women, and the incidence of Zika-positive pregnant women was 186.1 per 100,000 Table 3.

**Table 3:** Incidence rate of ZVD in pregnant women based on state, IMSS 2016.

		Suspected Cases		Positive cases	
State	Number of pregnant women in the period	Cases of ZVD in pregnant women	ZVD incidence in pregnant women per 100,000	Positive ZVD cases in pregnant women by RT-PCR	Positive Zika incidence in pregnant women per 100,000
Aguascalientes	11,319	0	0.0	0	0.0
Baja California	25,089	0	0.0	0	0.0
Baja California Sur	5,964	13	218.0	3	50.3
Campeche	3,821	173	4,527.6	36	942.2
Coahuila	24,974	2	8.0	1	4.0
Colima	5,247	204	3,887.9	27	514.6
Chiapas	9,283	597	6,431.1	170	1831.3
Chihuahua	26,639	2	7.5	0	0.0
Durango	9,058	1	11.0	0	0.0
Guanajuato	31,718	0	0.0	0	0.0
Guerrero	7,383	592	8,018.4	156	2113.0
Hidalgo	8,647	30	346.9	22	254.4
Jalisco	55,048	23	41.8	8	14.5
Mexico	61,999	5	8.1	2	3.2
Michoacán	17,264	12	69.5	2	11.6
Morelos	7,395	48	649.1	20	270.5
Nayarit	6,626	10	150.9	5	75.5
Nuevo León	41,372	288	696.1	102	246.5
Oaxaca	7,238	279	3,854.7	48	663.2
Puebla	16,420	8	48.7	1	6.1
Querétaro	14,899	1	6.7	0	0.0
Quintana Roo	12,803	97	757.6	40	312.4
San Luis Potosí	12,613	6	47.6	4	31.7
Sinaloa	17,786	34	191.2	10	56.2
Sonora	18,446	2	10.8	1	5.4
Tabasco	8,160	291	3,566.2	80	980.4
Tamaulipas	23,379	41	175.4	11	47.1
Tlaxcala	4,506	1	22.2	1	22.2
Veracruz	25,521	751	2,942.7	149	583.8
Yucatán	12,136	655	5,397.2	182	1499.7
Zacatecas	7,187	0	0.0	0	0.0
Mexico City	41,331	2	4.8	1	2.4
**Total IMSS**	**581,271**	**4,168**	**717.0**	**1,082**	**186.1**

The states with the highest incidence of positive ZVD cases in pregnant women were Guerrero, Chiapas and Yucatán Table 3.

## Discussion

As in other countries of the American continent, in Mexico, Zika virus spread during 2016. In total, 43,725 suspected cases of ZVD were reported to the IMSS, of which 1,700 (3.9%) were confirmed by PCR in the random sample (although serum samples were not collected from all suspected cases for laboratory analysis, as in other countries) [22]. These findings are similar to those in Colombia, which had 4% of cases confirmed in the same year [22]. The overall incidence of suspected ZVD cases was 82.0 cases per 100,000 inhabitants. The overall positive case estimate rate was 25.3 per 100,000—much higher than that previously reported (5.9 per 100,000) [23].

The incidence rates of ZVD were higher in women compared to those in men, and these findings are similar to those observed in Colombia [22]. The incidence rate of suspected cases of ZVD in nonpregnant women exceeded 13.1 per 100,000, which is higher to that of men. The difference in confirmed cases was more evident based on sex; in a previous study of confirmed cases of Zika in Mexico, 63% of women versus 37% of men were confirmed [24]. In total, 88.2% of confirmed cases were women, given that the epidemiological surveillance system covers a higher percentage of pregnant women compared with the general population. This is a bias of the study; differences were reduced by stratifying the analysis without pregnant women, and the proportion of positive ZVD cases in nonpregnant women was 1.76% and 1.26% in men. Among pregnant women, the cumulative positive ZVD incidence rates were the highest in Guerrero, Chiapas and Yucatán, with considerable geographical variation, and we found higher incidence levels than have been reported by previous estimates [13].

Regarding clinical manifestations, the most frequent symptoms were exanthema, pruritus, headache, myalgia and conjunctivitis, and these findings are consistent with other studies [25–26].The highest incidence rate occurred in men and women between 20 and 24 years of age, which reinforces the magnitude of infection and demands the attention of the health services for this economically active population. Other studies have shown that an increase in age is associated with an increased risk of suffering greater Zika symptomatology [24], suggesting that preexisting antibodies from a prior Dengue virus infection may enhance ZIKV infection [27].

In total, 1.7% of suspected cases of Zika were hospitalized, and a similar proportion was noted in Puerto Rico (2.0%) [28]. An association was found between hospitalization for suspected ZVD cases and diabetes, hypertension and pregnancy. Previously, a significant rise was observed in the hospitalization rate for congenital malformations of the nervous system, including Guillain-Barré syndrome, encephalitis, myelitis, and encephalomyelitis [29]. However, we did not find previous references to hospitalization of ZVD cases due to morbidities, such as diabetes, hypertension and pregnancy, found in this study. One of the limitations of the present study involved the inability to determine the route of transmission of the infection. Another limitation is that the epidemiological surveillance system of ZVD does not involve monitoring women during pregnancy, which allows for the identification of any fetal alterations.

The highest incidence of ZVD occurred in the southern states of the country, which is consistent with previously published data [13]. The altitude and climatic gradient add to the presence and abundance of *Ae*. *aegypti* in this region of Mexico [30]. Other ecologic factors, such as macroclimates and microclimates, are important to populations of mosquito species, such as *Ae*. *Aegypti* [31].

The highest incidence of ZVD was observed in the month of September (weeks 36–39), deriving from weather conditions, such as rainfall and environmental temperature, as other studies about dengue have shown in Mexico[32]. In September, the season is late summer and early fall, and it experiences more fluvial precipitation and higher temperatures than the rest of the year. In September 2016, Mexico registered 128.8 millimeters of fluvial precipitation and an average temperature of 25 degrees Celsius [33].

An important challenge for laboratory-based epidemiological surveillance of vector-borne diseases is the use of triplex diagnostic tests that assess dengue, chikungunya and Zika. Such tests were not available to the surveillance system during this study period. However, this test was incorporated as of November 2017 to obtain a differential diagnosis; in our country, these three vector-transmitted disease are present, and coinfections exist [34].

This report only includes symptomatic cases of ZVD, and current methods to identify and quickly manage these epidemics are limited by the brief window of diagnosis by PCR in acute infection. There is evidence that 80% of Zika cases can even be asymptomatic[26], although the most recent studies suggest that the percentage of asymptomatic Zika cases may be less than 30% [35]. Thus, the actual number of Zika cases should be much higher. In pregnant women, asymptomatic infections are likely associated with lower viremia, and even low levels of viremia can lead to congenital Zika syndrome [36]. We also share the idea that epidemiological surveillance in pregnant women based on rashes or other symptoms is not sufficient to determine exposure to Zika infection in areas or countries where Zika virus is circulating[37–38].

Of the total of suspicious cases with international travel three weeks prior to the onset of symptoms to zones where ZIKV circulates, 15.4% (2/13) were positive by PCR; this does not show that two cases were infected during the trip but confirms the significant role of surveillance networks for returning travelers to further enhance global disease surveillance [37].

This is the first report of Zika in Mexico using epidemiological surveillance that includes suspected cases, confirmed cases and laboratory positivity in a social security population, and it provides information on the magnitude of the ZIKV infection epidemic in Mexico.

In 2018, there has been a decrease in the number of suspected Zika cases in Mexico. This phenomenon has been observed in all countries of Latin America, suggesting that high immunity against further infection limits the capacity for ZIKV transmission. Although this had little impact on the eventual depletion of susceptible populations [38], it is likely that Zika will return when there is an increase in the susceptible population, as with dengue. The natural dynamics of the epidemic are likely to provide a multiyear window to develop new interventions before further large-scale outbreaks occur [39].

The epidemiological surveillance of arboviruses must continue to include Zika, since new epidemics such as yellow fever or Mayaro virus may also occur. At the beginning of October 2018, in Mexico the proportion of serious cases of dengue reached 63% compared to nonserious dengue cases. In the same week of 2017, cases of severe dengue increased by 53%, and deaths from dengue increased by 29% [40]; this phenomenon could be related to previous infection by Zika. As indicated in other studies, these data are timely and highly relevant from a public health standpoint given that a majority of regions currently experiencing Zika virus epidemics are also endemic for dengue [41–42]. This is an ongoing debate, as there is evidence that the existence of prior dengue antibodies is not related to the severity of Zika disease [43]; however, the inverse association has not been studied, i.e., an association between previous infection by Zika and the severity of dengue.

## Supporting information

S1 Fig Algorithm of laboratory sample in the epidemiological surveillance of Zika, in nonpregant persons, Mexico 2016. (TIF)

S1 File Characteristics of Zika cases in the Mexican Institute of Social Security, 2016 https://figshare.com/articles/Zika_virus_epidemiological_surveillance_of_the_Mexican_Institute_of_Social_Security/7502027. (XLSX)
