# Supplementary figures and images for: Zika virus: Epidemiological surveillance of the Mexican Institute of Social Security
Source: PLoS One. 2019 Feb 11;14(2):e0212114. doi: 10.1371/journal.pone.0212114 (PMC6370238; doi:10.1371/journal.pone.0212114)

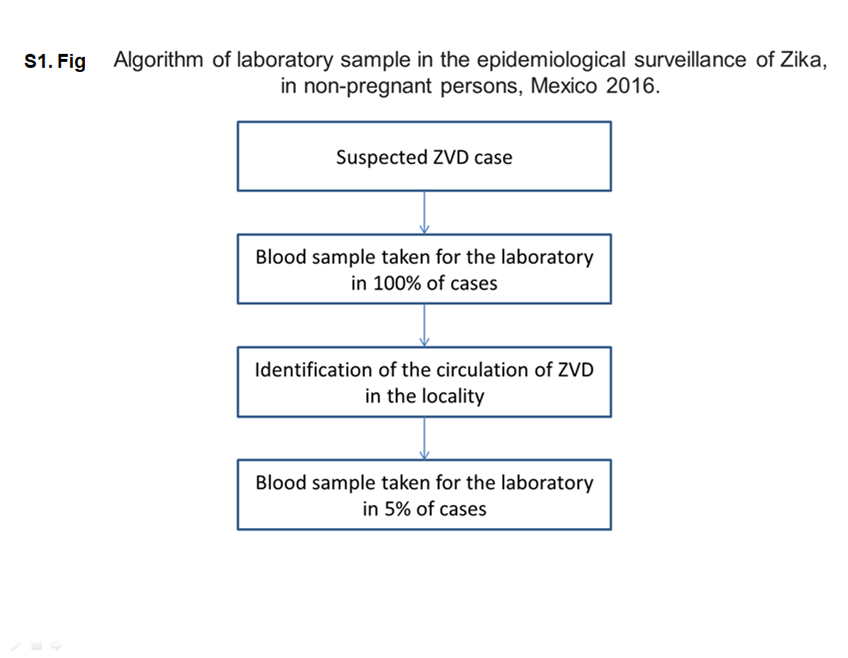

Supplement: S1 Fig — (TIF) [file pone.0212114.s001.tif]
